# A proposed FAIR approach for disseminating geospatial information system maps

**DOI:** 10.1038/s41597-023-02281-1

**Published:** 2023-06-16

**Authors:** P. Travis Thompson, Sweta Ojha, Christian D. Powell, Kelly G. Pennell, Hunter N. B. Moseley

**Affiliations:** 1 University of Kentucky Superfund Research Center (UKSRC), Lexington, KY USA; 2 University of Kentucky, College of Engineering, Department of Civil Engineering, Lexington, KY USA; 3 University of Kentucky, Department of Computer Science (Data Science Program), Lexington, KY USA; 4 University of Kentucky, Department of Molecular and Cellular Biochemistry, Lexington, KY USA

**Keywords:** Research data, Geography, Environmental impact

## Abstract

We present a draft Minimum Information About Geospatial Information System (MIAGIS) standard for facilitating public deposition of geospatial information system (GIS) datasets that follows the FAIR (Findable, Accessible, Interoperable and Reusable) principles. The draft MIAGIS standard includes a deposition directory structure and a minimum javascript object notation (JSON) metadata formatted file that is designed to capture critical metadata describing GIS layers and maps as well as their sources of data and methods of generation. The associated miagis Python package facilitates the creation of this MIAGIS metadata file and directly supports metadata extraction from both Esri JSON and GEOJSON GIS data formats plus options for extraction from user-specified JSON formats. We also demonstrate their use in crafting two example depositions of ArcGIS generated maps. We hope this draft MIAGIS standard along with the supporting miagis Python package will assist in establishing a GIS standards group that will develop the draft into a full standard for the wider GIS community as well as a future public repository for GIS datasets.

## Introduction

FAIR (Findable, Accessible, Interoperable and Reusable) is a set of guiding principles for managing publicly accessible data that emphasizes making data findable with unique identifiers and search mechanisms, easily accessible, i.e. downloadable, interoperable between computer systems and programs, and reusable for future use-cases^1,2^. For around a decade, ideas of FAIR-like stewardship have been implemented by several stakeholders^3^, especially to implement infrastructure that enables machine-actionable curation, conversion, and reuse of data^1,4^. Moreover, useful data, information, and knowledge derived from research should be shared in a FAIR manner that is transparent and usable by other researchers in related fields^4^. This extension of the FAIR principles moves closer to implementing the concept of open science by creating a research ecosystem that makes all research products FAIR for related researchers and stakeholders^5^.

However, the implementation of open science is a work in progress that requires the development of transparent procedures for depositing all types of research data and associated metadata. These procedures have been developed and codified for certain types of data, for example macromolecular structure in the Protein Data Bank^6^, genome sequence in the Sequence Read Archive, gene expression data in the Gene Expression Omnibus^7^, metabolomics data in Metabolomics Workbench^8^, and biological assay study data in BioStudies, for any biological assay modality^9^. These procedures include minimum reporting standards like MIAME, minimum information about a microarray experiment^10^, even though such minimum reporting standards have limitations^11^. Even with minimum standards, deposition quality, especially in terms of metadata needed to support reusability, is a constant issue that hinders FAIRness^12–14^. For research data types that have no procedures or minimum standards, these problems are far worse. Therefore, development of FAIR-minded procedures and minimum deposition standards and procedures are the first required steps towards bringing these data types into open science.

**Fig. 1:**
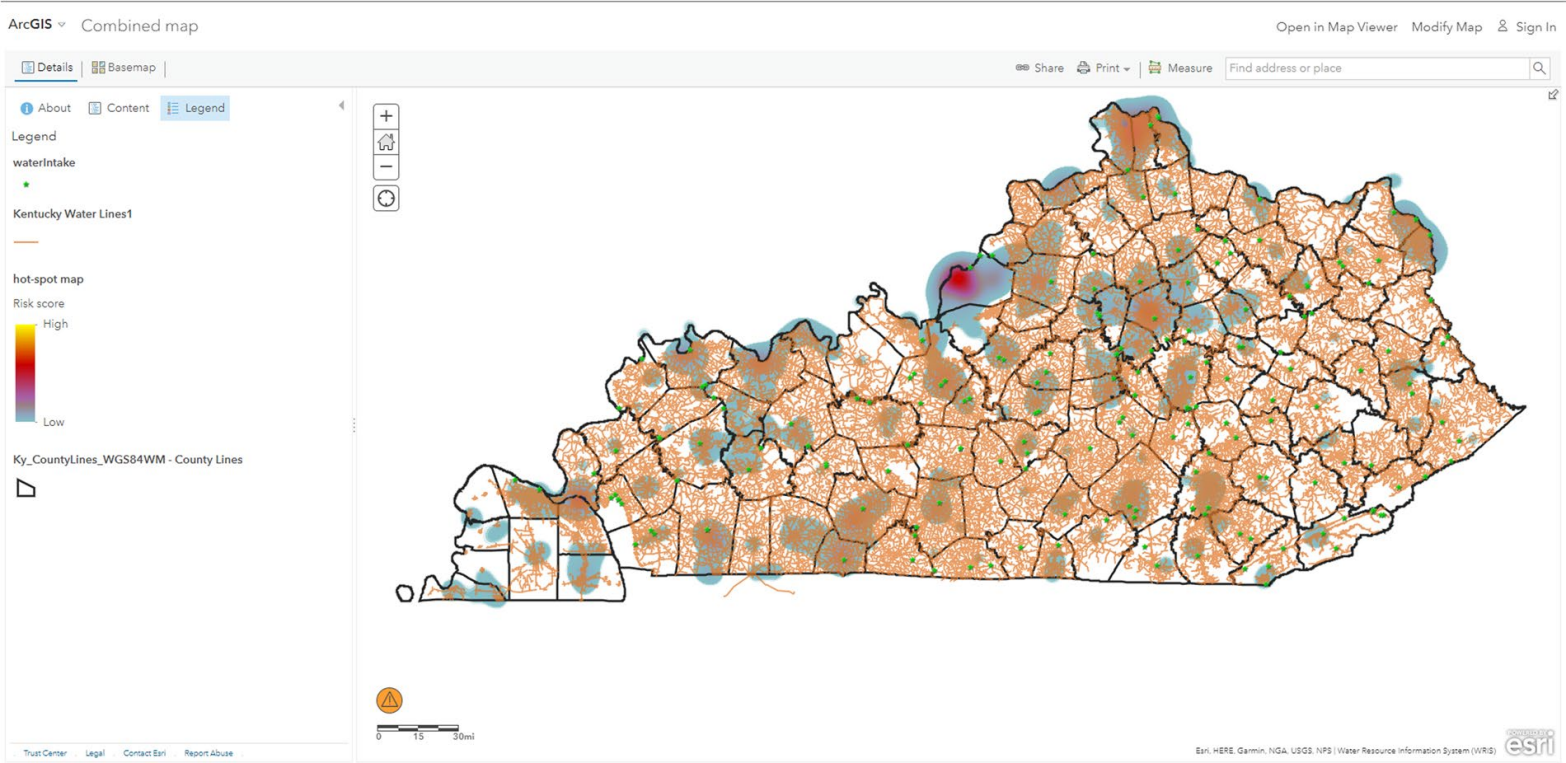
ArcGIS map example^33^.

A geospatial information system (GIS) is software for managing, organizing, and creating visual representations (maps) of data and information that are associated with specific geographical locations (geolocations). An example GIS map for the state of Kentucky is shown in Fig. 1.

GIS maps visualize any given type of data or information associated with specific geolocations. Thus, GIS datasets are composed of pairs with an arbitrary datum and its corresponding geolocation, where the first half of the pair is data type agnostic (i.e., supporting any data type). This makes GIS datasets and software very flexible with respect to the type of data being visualized. However, this data agnostic flexibility complicates deposition of GIS datasets into public scientific repositories, especially since no such open-deposit public repository currently exists for depositing GIS datasets in a FAIR manner by anyone in the broader GIS research community.

The ArcGIS Online mapping and analytics software is a very popular web-based GIS platform for generating and sharing GIS layers and maps^15^. However, ArcGIS layers and maps can be deleted by the user and the platform is thus not a true data repository providing dataset permanence. In addition, the ArcGIS platform is developed, maintained, and marketed by Esri, a software company headquartered in Redlands, California. An ArcGIS public account is free, but has limited non-commercial usage. Thus, ArcGIS must be licensed for full access. Also, ArcGIS does not provide unique persistent identifiers for datasets, like a Gene Expression Omnibus accession number id. Moreover, the ArcGIS object-relational database formats are proprietary. Fortunately, one of the ArcGIS map formats, Esri JSON, is in the highly computer parsable JavaScript Object Notation format; however, even this JSON format is only interoperable with some mapping and visualization tools, like open source QGIS^16^. While robust ecosystems of software tools have been developed to analyse geographical data and information, for example ArcGIS and QGIS, no open-deposit public repository exists for depositing such datasets in a FAIR manner by anyone in the broader GIS community. This is partly due to the development of this research community largely from civic purpose with a variety of government entities at the country, state, county, and even city level. As a government function, many GIS maps are continually maintained by these government entities in specialized GIS repositories^17,18^ without the concerns for permanence and FAIRness for the broader research enterprise. The presence of many closed and minimally FAIR GIS repositories along with the challenges of following FAIR has hindered the development of an open-deposit public GIS repository for the broader GIS community^17,19^. This lack of an open-deposit public GIS repository will become an acute problem for many researchers trying to comply with new government policies requiring public deposition of GIS datasets generated from federal grant funding, for example the new National Institutes of Health Data Management and Sharing Policy that went into effect January 25, 2023^20^.

Since no open-deposit public GIS repository exists, we have developed a draft Minimum Information About Geospatial Information System (MIAGIS) with associated deposition procedures for maximizing the FAIRness of ArcGIS analysed datasets and associated GIS maps. We demonstrate the application of MIAGIS and the deposition procedure to the generation of depositions for two separate sets of ArcGIS generated maps. Our goal is to kickstart the development of a FAIR deposition standard for GIS datasets as well as a community-serviceable meta repository^14^ for improving the Findability of GIS datasets, which could be trivially adapted for QGIS generated datasets as well. This grass roots approach has reached a level of success for other research communities^21^.

## Results

**Fig. 2:**
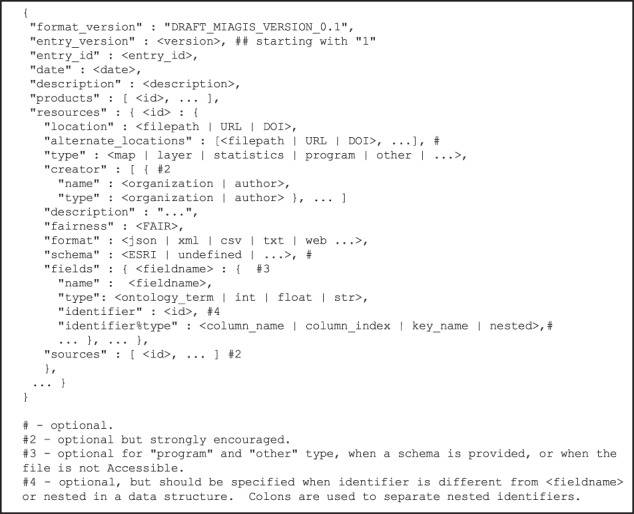
MIAGIS JSON data schema.

The draft MIAGIS standard is described in terms of a JSON data schema for a GIS metadata deposition file as shown in Fig. 2 along with a deposition directory structure illustrated in Fig. 5. The data schema provides a comprehensive description of the deposition that is computer parsable with a level of human readability. The deposition directory structure provides a consistent organization of GIS layers/maps along with optional source data. The first part of the data schema captures general entry metadata like MIAGIS format version, entry id, and entry description. The second part of the data schema contains “products” and “resources”. The heart of the data schema is the “resources” section, which captures the bulk of the description of a single data resource associated with a unique identifier along with an optional list of “sources”. The “products” and “sources” are simply lists of id’s, with each id referring back to a specific resource. Taken together, the “resources” descriptions along with the “products” and “sources” references enables data provenance for the deposition.

This data schema allows for a product-specific directed acyclic graph representation (DAG) that describes a product and the sources used to generate the product. This DAG representation can capture complex GIS analyses describing the data and computational resources used to generate a given GIS map. The “fields” subsection of the “resources” data schema was purposely designed to be very flexible with respect to data fields and their types to be applicable to the widest range of GIS datasets possible. Even some data structure nesting is allowed, but additional nesting flexibility may need to be designed into the full MIAGIS standard. In the current draft MIAGIS standard, all the top-level sections are required: “format_version”, “entry_version”, “entry_id”, “date”, “description”, “products”, and “resources”.

**Fig. 3:**
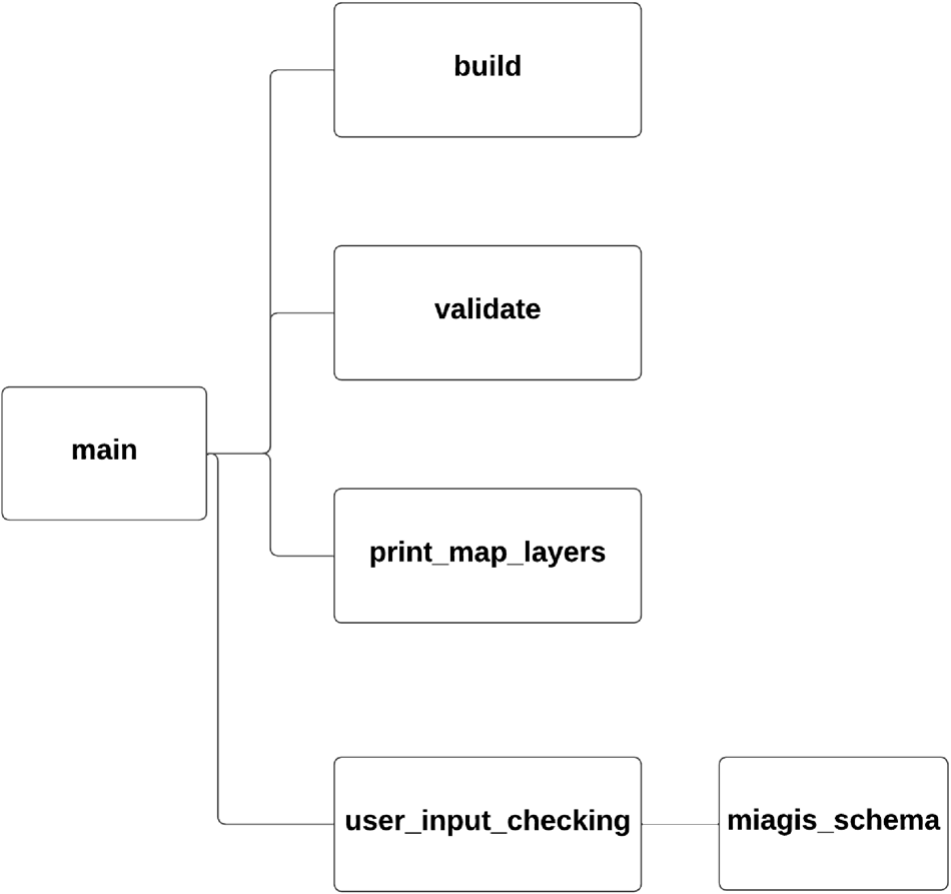
The miagis Python package module organization.

**Fig. 4:**
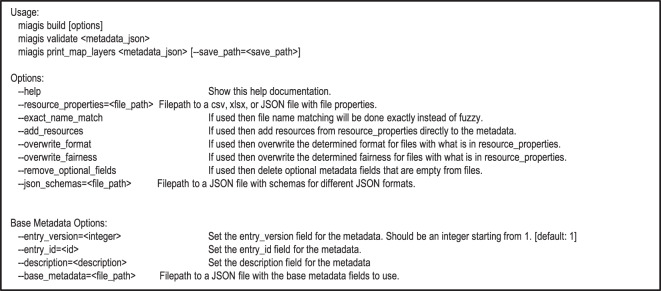
The miagis Python package command line interface.

Given the obvious burden to generate a MIAGIS-compliant deposition, we designed and implemented the miagis Python package to facilitate the generation of the MIAGIS-compliant JSON metadata file. To reduce confusion, we use lowercase miagis to refer to the Python software package and uppercase MIAGIS to refer to the deposition standard. The package organization is illustrated in Fig. 3 and provides the command line interface (CLI) shown in Fig. 4, which has three subcommands: “build”, “validate”, and “print_map_layers”.

**Fig. 5:**
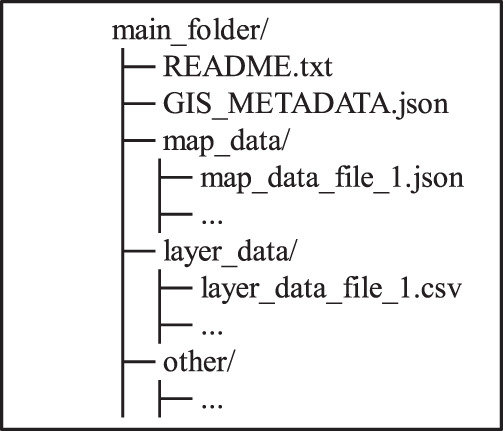
MIAGIS deposition directory structure.

**Fig. 6:**
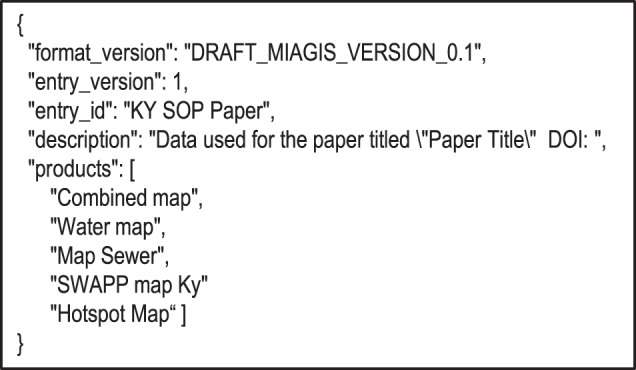
Example base metadata JSON file.

**Fig. 7:**
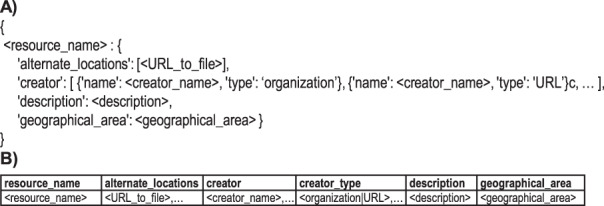
JSON (**A**) and tabular (**B**) representations of the resource properties file.

When given the “build” subcommand, the miagis package analyses a MIAGIS deposition directory structure (Fig. 5), parses relevant files, and generates a MIAGIS GIS_METADATA.json file. Several files are parsed including a base metadata file, a resource properties file, an optional JSON schemas file, and any GEOJSON and Esri JSON files identified in the resource properties file or via the deposition directory structure. The base metadata file is provided via the “–base_metadata” option and has the following sections: “format_version”, “entry_version”, “entry_id”, “description”, and “products”, with an example shown in Fig. 6. Unfortunately, much of this information must be filled out by hand because it cannot be determined or inferred programmatically. Therefore, it is best for the depositor/curator to provide this file so the build process can be easily redone with a minimum of repeated human intervention. The resource properties file is either a tabular or JSON file that contains information about resources in the deposition. Both JSON and tabular representations are shown in Fig. 7 and a full JSON example is shown in Supplementary Fig. 1. The JSON schemas file provides the miagis package a JSON schema description for additional JSON formatted files to parse beyond GEOJSON and Esri JSON. Next, the user can use the “validate” subcommand to validate the GIS_METADATA.json file and fix any discrepancies either through updating input files and redoing the build and/or editing the GIS_METADATA.json file directly with a text editor. The “print_map_layers” subcommand prints out the layered structure of the deposition as described in the GIS_METADATA.json file (e.g., see Supplementary Fig. 2).

MIAGIS, the deposition directory structure, and the miagis Python package were specifically designed to accommodate maps and layers generated by ArcGIS using the detailed deposition generation procedures in the Methods section. These procedures describe: (i) publishing ArcGIS layers, (ii) organizing the dataset under a consistent directory structure, (iii) creation of adequate metadata and description in draft MIAGIS JSON format, (iv) uploading the dataset to a general open data repository like Figshare^22^ or Zenodo^23^, (v) generating a Digital Object Identifier (DOI), and (vi) and cross-linking the public ArcGIS dataset with the public open repository as a stopgap measure for making the deposition Findable. With the exception of the first major step describing the generation of ArcGIS maps, these procedures can be directly adapted to handle GIS maps and layers generated by other GIS software, especially GIS maps and layers utilizing the GEOJSON format.

**Fig. 8:**
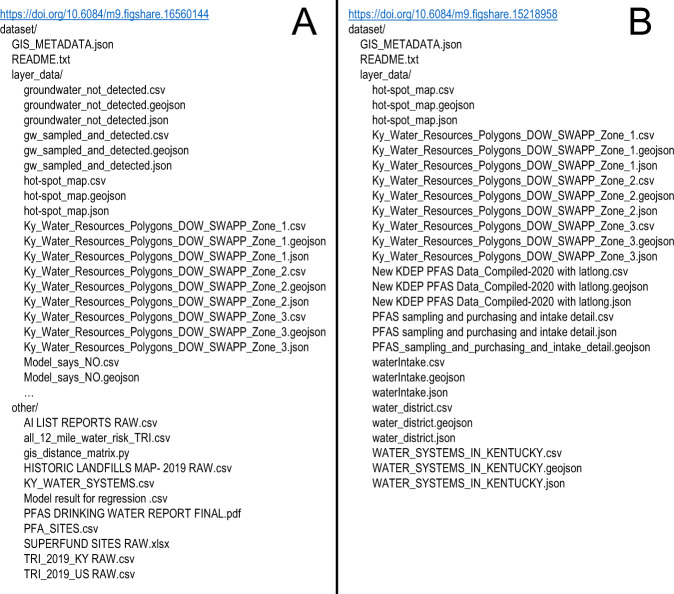
Two examples of draft MIAGIS-compliant depositions.

We have used these deposition generation procedures to craft two separate depositions publicly available on Figshare. Figure 8A shows the DOI and directory structure of a draft MIAGIS-compliant deposition for ArcGIS generated layers illustrating likely public water systems within the State of Kentucky where per- and polyfluoroalkyl substances (PFAS) are likely to be detected^24^. Figure 8B likewise shows the DOI and directory structure for a similarly constructed deposition for ArcGIS generated layers illustrating PFAS contamination in relation to potential PFAS users within the State of Kentucky^25^.

## Discussion

We have developed MIAGIS as a draft minimum deposition standard for GIS datasets with the associated miagis Python package for creating MIAGIS-compliant metadata in a JSONized deposition format. Furthermore, we have developed general procedures for converting ArcGIS layers/maps into a MIAGIS-compliant deposition. As demonstrated in our prior efforts^12,13,26^, the overall goal is for this combination of draft standard, tool, and procedures to both promote Open Science, specifically in the GIS community, as well as help reduce the burden of compliance to funding-source-specific research requirements like the new NIH Data Management and Sharing Policy. We have applied these procedures to two separate GIS dataset depositions that have utility to PFAS researchers and stakeholders within the State of Kentucky. Even with the description of MIAGIS, the deposition procedures, and the miagis Python package, it will take time for the members of the GIS community to become proficient enough to integrate these new procedures into their general workflow. We recommend that these procedures and the draft MIAGIS description be read carefully and any terms or steps that are unfamiliar should be looked up and understood. In the beginning, go slow and make sure every step is executed correctly before moving to the next step.

We hope the work presented here provides a starting point for the establishment of a GIS standards working group to further develop MIAGIS into a full standard for the GIS community. We expect an already established GIS standards organization like the Open Geospatial Consortium^27,28^ to create this MIAGIS standards working group. A natural first step, for this working group, is to incorporate relevant parts of the Content Standard For Digital Geospacial Metadata (CSDGM)^29^ developed by the Metadata Ad Hoc Working Group of the Federal Geographic Data Committee (FGDC) and further developed into the ISO 19115 Geographic Information Metadata Standard^30^, which is a minimum metadata content standard required for all federally generated GIS datasets. With this draft MIAGIS standard and a deposition SOP, we demonstrate the FAIRest deposition possible for publicly available maps generated within ArcGIS Online. But not all layers are necessarily FAIR. Some layers are generally Findable, but only interoperable and reusable within the ArcGIS ecosystem and not accessible. We label these layers as “Fir” within the GIS_METADATA.json file. This is an important distinction for data within restricted software ecosystems. While full FAIRness cannot be achieved in these situations, FAIRness can still be maximized. Technically, MIAGIS only improves: (i) Accessibility by providing the raw data of the map, which is not generally available through ArcGIS Online; (ii) Interoperability by providing descriptive metadata supporting data provenance in a machine readable JSON format, map data in machine readable formats, and a consistent directory organization of the deposition files; and (iii) Reusability by providing adequate data and metadata in machine readable formats. However, MIAGIS alone does not directly support Findability, which requires not only a persistent unique identifier (e.g. DOI) for a deposition, but also the systematic indexing of these identifiers in a manner that supports public searching. But we hope that the development of MIAGIS will enable the establishment of a community-serviceable meta repository for improving the Findability of GIS datasets that are Accessible from general public repositories like Figshare and Zenodo. So, either directly or indirectly, we see MIAGIS as a mechanism of improving GIS dataset FAIRness. Furthermore, we believe the associated deposition generation procedures can be trivially adapted for QGIS generated datasets as well.

## Methods

MIAGIS enables a generalized procedure for making geospatial data FAIRer. The major steps of this procedure are:

1. Make your map available to the public.
2. Create a metadata file for the underlying data.
3. Make the underlying data available on a general public repository.
4. Cross link the map and underlying data.

A detailed description for performing these steps is presented below.

### Procedure to publish layer publicly and share to group for an ArcGIS Online organizational account only

I. The layer is created and saved with proper name and tag.
II. After layer is saved, select Details > Content and click the ellipsis (three dots) that bring up **Show item details**’.
III. The layer detail will be shown and select ‘**Publish**’ that is situated on the right side of the details page. Proper name and tag should be selected to publish the layer.
IV. When the layer is published, ‘Share’, ‘Update’ and ‘Export’ options will be seen on the right side of the layer details page.
V. Click on ‘**Share**’ and select ‘Everyone (public)’ to share the layer to public.
VI. Click ‘**Edit group sharing**’ from the bottom of the sharing option page and select groups to allow their members to export and download the data of the layer.
VII. The last option is to select ‘Settings’ option from top right side of the layer details page and select ‘Allows others to export to different formats’ that is situated on the bottom of the Settings page.

Note: The owner can only publish and share the layer if the owner created the layer by himself or herself, making the layer FAIR, but with no permanence. The group members to whom the published layers were shared can extract or download the data in different forms, but only group members. However, if the owner extracted the layer from another source, it can be shared but its data cannot be downloaded or extracted from the layer, making it generally Findable but only interoperable and reusable within the ArcGIS ecosystem and not accessible (downloadable). In other words, the layer is “Fir”, where lowercase indicates the restriction to the closed software ecosystem. This inability to allow the public to download layer data is one reason why a public repository must be used and linked to the layer to achieve a better level of FAIRness. A user can get the layer data from the repository, and the draft MIAGIS standard describes where the data is located for each layer.

### Procedures for creating a draft MIAGIS-compliant deposition and making it publicly available on Figshare

ArcGIS Online lacks the ability to create Digital Object Identifiers (DOIs), so this feature is outsourced to Figshare. But first, you will need to make an account with Figshare. Then a Figshare deposition item can be created as follows:

- On your computer create a folder to hold all the data, including maps, layers, and any other data.
- Within that folder, create the following subfolders as needed: ‘map_data/’, ‘layer_data/’, and ‘other/’. Note that the forward slash indicates a subfolder on Mac and Linux operating systems, while a back slash indicates a subfolder on Windows.
- Place whole maps (integrated collection of layers) into the ‘map_data/’ subfolder.
- Place the processed layer data (data used to create feature layers) in the ‘layer_data/’ subfolder.
- Place any other data or files in the ‘other/’ subfolder.
- Create a README.txt (or README.md) file to be included in the main folder which provides a description of the datasets included and a copy of any relevant license agreements.

- Add a URL to the dataset on ArcGIS Online into this README file.
- README.md is a Markdown text file that will allow formatted visualization in a browser.

g. Create a MIAGIS-compliant GIS_METADATA.json file that will contain metadata and a description of all files and data sources.
h. The file directory should now look like this:

i. Compress the main folder to create an archive (i.e., ZIP, TAR.GZ, etc.).
j. In the ‘My data’ menu, click ‘**+Create a new item**’.
k. In the new item window, drag and drop the compressed archive or click browse to upload the datasets to the Figshare item.
l. Fill out the remainder of the fields in the new item window.
m. In the new item window, click ‘**Reserve Digital Object Identifier**’. This will create a single DOI that can be used for the datasets.
n. In the new item window, click ‘**Save changes**’.
o. The item can be edited by reopening the new item window by clicking on the item in the “My data” menu. After editing the data, follow the process below:

i. Reopen the added item window by clicking on the item in the “My data” menu.
ii. In the item window, make sure Publish is selected.
iii. In the item window, click ‘**Save changes**’.
iv. The data of the Figshare item can be updated and all updated versions can be seen in the Figshare item; for example, the Figshare item for Ojha *et al*.^24^, with the following 10.6084/m9.figshare.16560144^31^. Multiple version updates can be seen in this Figshare item.

### Creating MIAGIS-compliant metadata JSON file

Figure 2 illustrates the draft MIAGIS JSON data schema. The helper miagis Python package is available to generate most of this metadata file, which can then be edited by the user. The miagis package can also validate the final JSON metadata file before inclusion in the Figshare item. The miagis Python package is written in Python 3.7, is pip installable, and has comprehensive end-user documentation with installation instructions for Linux, Mac, and Windows operating systems. The package has both a command line interface (CLI) and application programming interface (API). Therefore, data curators can directly use miagis via its CLI as well as incorporate miagis into deposition software pipelines via its API.

### Updating ArcGIS content to include a DOI

The description pages data files, feature layers, and story maps created from the datasets, which were uploaded to the Figshare item, should be updated to include the generated DOI.

- Sign into ArcGIS Online.
- From the main menu bar, click ‘**Content’** to show content items.
- Click on the desired data file, feature layer, or story map to update.
- In the content’s description, add the DOI.

- E.G.: “10.3390/metabo11030163”

e. Ensure that each data file, feature layer, and story map is published, and the sharing setting is set to public.

## Supplementary information

Supplementary Material

## Data Availability

A Figshare item titled “A Geospatial and Binomial Logistic Regression Model to Prioritize Sampling for Per- and Polyfluorinated Alkyl Substances (PFAS) in Public Water Systems-Dataset” which includes GIS layers derived from the results of a regression model. 10.6084/m9.figshare.16560144^31^. A Figshare item titled “A FAIR approach to detect and share PFAS hot-spot areas and water systems in Kentucky”, which includes GIS layers identifying PFAS hot-spot areas and water systems in Kentucky. 10.6084/m9.figshare.15218958^32^.

## Code availability

The miagis Python package is available on GitHub and the Python Package Index with comprehensive user documentation on GitHub.io:

https://github.com/MoseleyBioinformaticsLab/miagis

https://pypi.org/project/miagis/

https://moseleybioinformaticslab.github.io/miagis/
