# Supplementary material for: A proposed FAIR approach for disseminating geospatial information system maps
Source: Sci Data. 2023 Jun 16;10:389. doi: 10.1038/s41597-023-02281-1 (PMC10275873; doi:10.1038/s41597-023-02281-1)
Supplement: Supplementary file 1 — Supplementary Material [file 41597_2023_2281_MOESM1_ESM.docx]

**A proposed FAIR approach for disseminating geospatial information system maps**

*P. Travis Thompson^1^, Sweta Ojha^1,2^, Christian D. Powell^1 ,3^ Kelly G. Pennell^1,2^, and Hunter N.B. Moseley^1,4^**

^1^University of Kentucky Superfund Research Center (UKSRC)

^2^University of Kentucky, College of Engineering, Department of Civil Engineering

^3^University of Kentucky, Department of Computer Science (Data Science Program)

^4^University of Kentucky, Department of Molecular and Cellular Biochemistry

* Corresponding Author- [hunter.moseley@uky.edu](mailto:hunter.moseley@uky.edu)

{

'PFAS_sampling_and_purchasing_and_intake_detail': {

'alternate_locations': ['https://services.arcgis.com/vQ8kO5zdqETeirEL/arcgis/rest/services/PFAS_sampling_and_purchasing_data_2019/FeatureServer'],

'creator': [{'name': 'Kentucky Department of Environmental Protection',

'type': 'organization'},

{'name': 'https://eec.ky.gov/Documents%20for%20URLs/PFAS%20Drinking%20Water%20Report%20Final.pdf',

'type': 'URL'}],

'description': 'List of all water systems in which PFAS were sampled.',

'geographical_area': 'Kentucky'},

'Ohio_River_Marinas_WFL1 - Ohio River': {

'alternate_locations': ['https://services8.arcgis.com/Xcpl3GIMvkCI3oFI/arcgis/rest/services/Ohio_River_Marinas_WFL1/FeatureServer'],

'creator': [{'name': 'ArcGIS Online', 'type': 'organization'}],

'description': 'Publicly available layer findable on ArcGIS Online.',

'geographical_area': 'Kentucky'},

'Kentucky Water Lines1': {

'alternate_locations': ['https://kygisserver.ky.gov/arcgis/rest/services/WGS84WM_Services/Ky_Water_WGS84WM/MapServer/11',

'https://uky-edu.maps.arcgis.com/home/item.html?id=29713c2b8be14534943b8e2e5fa16daa'],

'creator': [{'name': 'https://kygeoportal.ky.gov/', 'type': 'URL'}],

'description': 'Locations of water lines in Kentucky.',

'geographical_area': 'Kentucky'}

}

**Supplementary Figure 1.** Full JSON example of the resource properties file.

Maps:

Map 1 Potential hot-spot map

Layers:

PFAS_detected_sites

PFAS_sampling_and_purchasing_and_intake_detail

TRI_DATA_IN_KENTUCKY

Risk map with landfill

Ohio_River_Marinas_WFL1 - Ohio River

Ky_Water_Resources_Polygons_DOW_SWAPP_Zone_2

Ky_Water_Resources_Polygons_DOW_SWAPP_Zone_1

Ky_Water_Resources_Polygons_DOW_SWAPP_Zone_3

waterIntake

Surfacewater_sampled_and_detected

gw_sampled_and_detected

groundwater_not_detected

Surface_water_sampled_and_not_detected

WATER_SYSTEMS_IN_KENTUCKY

water_district

Model_says_NO

hot-spot_map

Model_says_yes

Waste WTP outfls

Kentucky County Polygons

Blank White Vector Basemap

US map

Layers:

TRI_only_A

TRI_CA-HI

TRI__IA-LA

TRI__MA-MT

TRI__NC-OH

TRI__TX-WV

TRI__OK-TN

USA States Generalized1

USA_State_Internal_Boundaries

Blank White Vector Basemap

**Supplementary Figure 2.** Example output from the “print_map_layers” command of the miagis package.
